# Supplementary material for: Metabolic Status Influences Probiotic Efficacy for Depression—PRO-DEMET Randomized Clinical Trial Results
Source: Nutrients. 2024 May 3;16(9):1389. doi: 10.3390/nu16091389 (PMC11085729; doi:10.3390/nu16091389)
Supplement: Supplementary file 1 [file nutrients-16-01389-s001.zip › nutrients-2979804-supplementary.pdf]

1. Basal psychopathology scores in the PRO group depend on antidepressant treatment.

Aggregate Results
Descriptive Statistics (Spreadsheet2_(Recovered) 5.01.2024)
Include condition: v1="pro"

Variable	Antidepressants	Valid N	Mean	Minimum	Maximum	Std.Dev.
**V1 MADRS**	yes	36	20.16667	13.00000	43.0000	6.48735
V1 DASS	yes	35	58.97143	16.00000	100.0000	22.40468
V1 D-DASS	yes	35	18.65714	3.00000	37.0000	10.05848
V1 A-DASS	yes	35	16.88571	1.00000	38.0000	9.61240
V1 S-DASS	yes	35	23.42857	8.00000	42.0000	8.56561
V1 MADRS sadness	yes	32	4.34375	1.00000	7.0000	1.78902
V1 MADRS neurovegetative	yes	32	5.46875	0.00000	10.0000	2.24304
V1 MADRS detachment	yes	32	6.84375	0.00000	10.0000	2.47711
V1 MADRS negative thoughts	yes	32	3.00000	1.00000	8.0000	1.50269
V1 MADRS	no	15	22.80000	16.00000	29.0000	4.61674
V1 DASS	no	15	74.40000	37.00000	106.0000	17.92365
V1 D-DASS	no	15	25.60000	7.00000	40.0000	10.07685
V1 A-DASS	no	15	20.06667	4.00000	30.0000	6.88131
V1 S-DASS	no	15	28.73333	17.00000	37.0000	7.10600
V1 MADRS sadness	no	15	4.66667	2.00000	7.0000	1.44749
V1 MADRS neurovegetative	no	15	6.86667	2.00000	10.0000	2.13363
V1 MADRS detachment	no	15	7.66667	2.00000	11.0000	2.16025
V1 MADRS negative thoughts	no	15	3.53333	2.00000	5.0000	1.06010

Aggregate Results
Mann-Whitney U Test (w/ continuity correction) (Spreadsheet2_(Recovered) 5.01.2024)
By variable Antidepressants

variable	Probiotic/placebo	Rank Sum no	Rank Sum yes	U	Z	p-value	Z adjusted	p-value	Valid N no	Valid N yes	2*1sided exact p
**V1 MADRS**	plc	343.0000	603.0000	168.0000	0.894171	0.371231	0.898832	0.368743	14	29	0.375697
V1 DASS	plc	338.0000	565.0000	159.0000	0.973882	0.330116	0.974356	0.329881	14	28	0.334373
V1 D-DASS	plc	316.5000	586.5000	180.5000	0.400225	0.688991	0.400908	0.688488	14	28	0.682813
V1 A-DASS	plc	315.0000	588.0000	182.0000	0.360203	0.718696	0.361302	0.717874	14	28	0.721960
V1 S-DASS	plc	364.5000	538.5000	132.5000	1.680947	0.092774	1.682583	0.092457	14	28	0.090394
V1 MADRS sadnes	plc	343.0000	560.0000	154.0000	1.107290	0.268169	1.126958	0.259761	14	28	0.271830
V1 MADRS neurove	plc	323.5000	579.5000	173.5000	0.586997	0.557206	0.596598	0.550776	14	28	0.552761
V1 MADRS detachn	plc	318.5000	584.5000	178.5000	0.453589	0.650125	0.459185	0.646101	14	28	0.644482
V1 MADRS negative	plc	319.5000	583.5000	177.5000	0.480270	0.631035	0.491866	0.622815	14	28	0.625648
V1 MADRS	pro	847.0000	479.0000	181.0000	-1.82951	0.067324	-1.83304	0.066797	36	15	0.066949
V1 DASS	pro	790.0000	485.0000	160.0000	-2.15936	0.030823	-2.16025	0.030754	35	15	0.029708
V1 D-DASS	pro	792.0000	483.0000	162.0000	-2.11702	0.034259	-2.12110	0.033914	35	15	0.033193
V1 A-DASS	pro	829.5000	445.5000	199.5000	-1.32314	0.185790	-1.32610	0.184806	35	15	0.184383
V1 S-DASS	pro	802.0000	473.0000	172.0000	-1.90532	0.056739	-1.90849	0.056329	35	15	0.056118
V1 MADRS sadnes	pro	748.5000	379.5000	220.5000	-0.43361	0.664569	-0.44103	0.659190	32	15	0.659802
V1 MADRS neurove	pro	681.0000	447.0000	153.0000	-1.97408	0.048373	-1.99749	0.045773	32	15	0.047553
V1 MADRS detachn	pro	726.0000	402.0000	198.0000	-0.94710	0.343587	-0.95853	0.337795	32	15	0.347578
V1 MADRS negative	pro	698.0000	430.0000	170.0000	-1.58611	0.112715	-1.65371	0.098188	32	15	0.113415

2. Basal MADRS and DASS score, or antidepressant use in the PRO group depending on abdominal obesity or HSI>36 presence.

Aggregate Results
Mann-Whitney U Test (w/ continuity correction) (Spreadsheet2_(Recovered) 5.01.2024)
By variable WC 2022
Marked tests are significant at p <.05000

variable	Probiotic/placebo	Rank Sum no	Rank Sum yes	U	Z	p-value	Z adjusted	p-value	Valid N no	Valid N yes	2*1sided exact p
V1 MADRS	pro	389.0000	937.0000	236.0000	-1.04902	0.294169	-1.05104	0.293239	17	34	0.297401
V1 DASS	pro	434.0000	841.0000	280.0000	0.00000	1.000000	0.00000	1.000000	17	33	1.000000
V1 D-DASS	pro	444.5000	830.5000	269.5000	0.21504	0.829739	0.21545	0.829416	17	33	0.823568
V1 A-DASS	pro	472.0000	803.0000	242.0000	0.77823	0.436434	0.77997	0.435407	17	33	0.440305
V1 S-DASS	pro	364.0000	911.0000	211.0000	-1.41310	0.157627	-1.41545	0.156938	17	33	0.159124
V1 MADRS sadness	pro	364.5000	763.5000	211.5000	-0.95204	0.341079	-0.96832	0.332883	17	30	0.339135
V1 MADRS neurovegetative	pro	379.5000	748.5000	226.5000	-0.61993	0.535304	-0.62728	0.530475	17	30	0.531909
V1 MADRS detachment	pro	384.0000	744.0000	231.0000	-0.52030	0.602856	-0.52658	0.598487	17	30	0.606414
V1 MADRS negative thoughts	pro	365.5000	762.5000	212.5000	-0.92990	0.352426	-0.96952	0.332285	17	30	0.350427

Aggregate Results
Mann-Whitney U Test (w/ continuity correction) (Spreadsheet2_(Recovered) 5.01.2024)
By variable HSI>36 yes/no
Marked tests are significant at p <.05000

variable	Probiotic/placebo	Rank Sum no	Rank Sum yes	U	Z	p-value	Z adjusted	p-value	Valid N no	Valid N yes	2*1sided exact p
V1 MADRS	pro	1075.500	250.5000	195.5000	0.213520	0.830921	0.213932	0.830600	41	10	0.824420
V1 DASS	pro	1021.500	253.5000	198.5000	0.024254	0.980650	0.024263	0.980642	40	10	0.971440
V1 D-DASS	pro	1005.500	269.5000	185.5000	-0.339550	0.734196	-0.340204	0.733703	40	10	0.729117
V1 A-DASS	pro	1005.500	269.5000	185.5000	-0.339550	0.734196	-0.340311	0.733623	40	10	0.729117
V1 S-DASS	pro	1067.500	207.5000	152.5000	1.139917	0.254322	1.141811	0.253534	40	10	0.253646
V1 MADRS sadness	pro	873.000	255.0000	170.0000	-0.376910	0.706241	-0.383358	0.701454	37	10	0.710533
V1 MADRS neurovegetative	pro	885.000	243.0000	182.0000	-0.064984	0.948186	-0.065755	0.947573	37	10	0.949014
V1 MADRS detachment	pro	880.000	248.0000	177.0000	-0.194953	0.845430	-0.197306	0.843588	37	10	0.847846
V1 MADRS negative thoughts	pro	913.000	215.0000	160.0000	0.636847	0.524225	0.663987	0.506699	37	10	0.530037

Probiotic/placebo	Antidepressants	HSI>36 yes/no no	HSI>36 yes/no yes	Row Totals
plc	no	13	1	14
plc	yes	17	13	30
plc	All Grps	30	14	44
pro	yes	9	27	36
pro	no	1	14	15
pro	All Grps	10	41	51

PRO: The chi-square statistic is 2.2577. The *p*-value is .132953. The result is *not* significant at $p < .05$.

Include condition: v1="pro"

WC 2022	Antidepressants no	Antidepressants yes	Row Totals
no	12	22	34
yes	3	14	17
All Grps	15	36	51

The chi-square statistic is 1.7. The *p*-value is .192288. The result is *not* significant at $p < .05$.

3. Correlation analysis of basal psychometric and metabolic data.

All Groups
Correlations (Spreadsheet2_(Recovered) 5.01.2024)
Marked correlations are significant at p < .05000
N=87 (Casewise deletion of missing data)

Variable	**V1 MADRS**	V1 DASS	V1 D-DASS	V1 A-DASS	V1 S-DASS	V1 MADRS sadness	V1 MADRS neurovegetative	V1 MADRS detachment	V1 MADRS negative thoughts
**V1 BMI [kg/m2]**	-0.001363	0.058847	0.178982	0.067991	-0.111485	0.020791	-0.038799	0.006601	-0.055551
V1 WC [cm]	0.003885	0.046282	0.132750	0.093090	-0.116433	0.028398	-0.093595	0.074386	-0.121303
V1 AST	0.079293	-0.144101	-0.096126	-0.154609	-0.101983	0.169614	-0.012366	0.020457	0.106702
V1 ALT	-0.025974	-0.150218	-0.094121	-0.118246	-0.153173	0.112573	-0.066820	-0.076352	-0.029680
AST/ALT	0.079293	-0.144101	-0.096126	-0.154609	-0.101983	0.169614	-0.012366	0.020457	0.106702
V1 sBP [mmHg]	0.094618	-0.091041	-0.017454	-0.048963	-0.156284	0.079280	0.007670	0.001165	0.109853
V1 dBP [mmHg]	0.187061	0.012286	0.069421	0.074861	-0.114438	0.187498	0.026995	0.097349	0.206173
V1 fGlc [mmol/l]	0.176602	0.004089	0.112830	0.018364	-0.127325	0.089343	0.135160	0.125448	0.030290
V1 TG	-0.011872	-0.051887	0.104647	-0.090882	-0.151664	0.101852	-0.076864	0.021208	-0.165300
V1 TG [mg/dl]	-0.011872	-0.051887	0.104647	-0.090882	-0.151664	0.101852	-0.076864	0.021208	-0.165300
V1 HDL [mmol/l]	-0.305562	-0.127200	-0.282654	-0.069396	0.057183	-0.245066	-0.178171	-0.257789	-0.205326
V1 TG/HDL	0.079659	-0.026098	0.160624	-0.072428	-0.165971	0.178894	0.022260	0.057470	-0.087516
V1 non-HDL-c	-0.097574	-0.122864	-0.069591	-0.059484	-0.168169	-0.044565	-0.075497	-0.049491	-0.177625
V1 LDL-c	-0.126316	-0.120944	-0.119775	-0.038991	-0.129431	-0.098730	-0.072403	-0.067617	-0.180817
V1 cholesterole	-0.181651	-0.151977	-0.148844	-0.075907	-0.139090	-0.114408	-0.123190	-0.122772	-0.226100
HSI	-0.066705	-0.003148	0.082767	0.036478	-0.129964	-0.020390	-0.057335	-0.051853	-0.122341

Aggregate Results
Correlations (Spreadsheet2_(Recovered) 5.01.2024)
Marked correlations are significant at p < .05000

Variable	**Probiotic/placebo**	V1 MADRS	V1 DASS	V1 D-DASS	V1 A-DASS	V1 S-DASS	V1 MADRS sadness	V1 MADRS neurovegetative	V1 MADRS detachment	V1 MADRS negative thoughts
**V1 BMI [kg/m2]**	plc	0.114476	0.191927	0.341678	0.176365	-0.026423	0.091110	0.152837	0.034062	0.020174
V1 WC [cm]	plc	0.136540	0.187850	0.320759	0.205906	-0.042009	0.128560	0.125809	0.162205	-0.093183
V1 AST	plc	0.219025	-0.053149	0.034452	-0.129984	-0.042217	0.244923	0.012607	0.105300	0.263112
V1 ALT	plc	0.222862	-0.016192	0.108850	-0.067545	-0.079957	0.253056	0.030347	0.165906	0.159047
AST/ALT	plc	0.219025	-0.053149	0.034452	-0.129984	-0.042217	0.244923	0.012607	0.105300	0.263112
V1 sBP [mmHg]	plc	0.253218	0.173911	0.248669	0.107476	0.077946	0.112728	0.207824	0.165201	0.211281
V1 dBP [mmHg]	plc	0.422146	0.188623	0.295621	0.231987	-0.039475	0.355978	0.252578	0.209669	0.346663
V1 fGlc [mmol/l]	plc	0.280448	0.066791	0.159928	0.161648	-0.134115	0.129420	0.268383	0.226251	0.118028
V1 TG	plc	0.108823	0.009373	0.207943	-0.043727	-0.133495	0.134614	0.170869	0.032624	-0.078081
V1 TG [mg/dl]	plc	0.108823	0.009373	0.207943	-0.043727	-0.133495	0.134614	0.170869	0.032624	-0.078081
V1 HDL [mmol/l]	plc	-0.357858	-0.121098	-0.272136	-0.103294	0.062249	-0.302135	-0.302886	-0.145895	-0.218930
V1 TG/HDL	plc	0.215484	0.034067	0.248257	-0.028947	-0.126658	0.241164	0.234295	0.081186	-0.005702
V1 non-HDL-c	plc	-0.215075	-0.184826	-0.046783	-0.124078	-0.276187	-0.212940	-0.003539	-0.109350	-0.298117
V1 LDL-c	plc	-0.283154	-0.205731	-0.114430	-0.117644	-0.267272	-0.287381	-0.080445	-0.114571	-0.322713
V1 cholesterole	plc	-0.298031	-0.209504	-0.113405	-0.146145	-0.252257	-0.282030	-0.079159	-0.142514	-0.343823
HSI	plc	0.085528	0.131121	0.256009	0.149663	-0.064278	0.053676	0.115415	0.066596	-0.023959

Aggregate Results
Correlations (Spreadsheet2_(Recovered) 5.01.2024)
Marked correlations are significant at p < .05000

Variable	**Probiotic/placebo**	V1 MADRS	V1 DASS	V1 D-DASS	V1 A-DASS	V1 S-DASS	V1 MADRS sadness	V1 MADRS neurovegetative	V1 MADRS detachment	V1 MADRS negative thoughts
V1 BMI [kg/m2]	pro	-0.081995	-0.102457	0.004929	-0.033122	-0.232958	-0.063713	-0.152071	-0.039495	-0.154865
V1 WC [cm]	pro	-0.089180	-0.112694	-0.053832	-0.008746	-0.214096	-0.085651	-0.241847	-0.011875	-0.162108
V1 AST	pro	-0.108573	-0.361053	-0.353310	-0.242727	-0.253639	0.037959	-0.061266	-0.107158	-0.182698
V1 ALT	pro	-0.226538	-0.286130	-0.270664	-0.170805	-0.233942	-0.042649	-0.165719	-0.271218	-0.219937
AST/ALT	pro	-0.108573	-0.361053	-0.353310	-0.242727	-0.253639	0.037959	-0.061266	-0.107158	-0.182698
V1 sBP [mmHg]	pro	-0.021576	-0.336391	-0.226296	-0.189498	-0.395468	0.045260	-0.158604	-0.115235	0.018316
V1 dBP [mmHg]	pro	0.036963	-0.129068	-0.083320	-0.048385	-0.180579	0.039897	-0.136189	0.030738	0.095226
V1 fGlc [mmol/l]	pro	0.113218	-0.060743	0.066616	-0.107345	-0.124593	0.048404	0.065970	0.044054	-0.057923
V1 TG	pro	-0.081926	-0.104991	0.026339	-0.124145	-0.172401	0.074333	-0.225562	0.008430	-0.239500
V1 TG [mg/dl]	pro	-0.081926	-0.104991	0.026339	-0.124145	-0.172401	0.074333	-0.225562	0.008430	-0.239500
V1 HDL [mmol/l]	pro	-0.319284	-0.125387	-0.271741	-0.067484	0.071525	-0.226607	-0.212274	-0.300303	-0.196741
V1 TG/HDL	pro	0.002330	-0.083883	0.085087	-0.102148	-0.210650	0.123666	-0.089061	0.031465	-0.163709
V1 non-HDL-c	pro	-0.029825	-0.062889	-0.075753	-0.011640	-0.058429	0.116512	-0.165255	0.002294	-0.066664
V1 LDL-c	pro	-0.037283	-0.036686	-0.107353	0.018559	0.015059	0.084339	-0.124560	-0.019147	-0.046584
V1 cholesterole	pro	-0.130203	-0.097540	-0.156490	-0.032359	-0.029876	0.032443	-0.218440	-0.094943	-0.124017
HSI	pro	-0.165620	-0.131663	-0.064824	-0.054988	-0.202405	-0.093020	-0.156403	-0.149414	-0.218060

4. Correlation analysis of basal psychopathological data.

All Groups
Correlations (Spreadsheet2_(Recovered) 5.01.2024)
Marked correlations are significant at p < .05000
N=87 (Casewise deletion of missing data)

Variable	V1 MADRS	V1 DASS	V1 D-DASS	V1 A-DASS	V1 S-DASS	V1 MADRS sadness	V1 MADRS neurovegetative	V1 MADRS detachment	V1 MADRS negative thoughts
**V1 MADRS**	1.000000	0.547920	0.551889	0.499183	0.273044	0.694281	0.654911	0.639394	0.744665
V1 DASS	0.547920	1.000000	0.793989	0.837984	0.793827	0.333501	0.357401	0.478539	0.417237
V1 D-DASS	0.551889	0.793989	1.000000	0.509611	0.383852	0.433582	0.268519	0.442875	0.478631
V1 A-DASS	0.499183	0.837984	0.509611	1.000000	0.550636	0.290668	0.378021	0.410211	0.341394
V1 S-DASS	0.273044	0.793827	0.383852	0.550636	1.000000	0.074738	0.226052	0.304316	0.182297
V1 MADRS sadness	0.694281	0.333501	0.433582	0.290668	0.074738	1.000000	0.270668	0.309083	0.479207
V1 MADRS neurovegetative	0.654911	0.357401	0.268519	0.378021	0.226052	0.270668	1.000000	0.129046	0.411684
V1 MADRS detachment	0.639394	0.478539	0.442875	0.410211	0.304316	0.309083	0.129046	1.000000	0.276887
V1 MADRS negative thoughts	0.744665	0.417237	0.478631	0.341394	0.182297	0.479207	0.411684	0.276887	1.000000

Aggregate Results
Correlations (Spreadsheet2_(Recovered) 5.01.2024)
Marked correlations are significant at p < .05000

Variable	Probiotic/placebo	V1 MADRS	V1 DASS	V1 D-DASS	V1 A-DASS	V1 S-DASS	V1 MADRS sadness	V1 MADRS neurovegetative	V1 MADRS detachment	V1 MADRS negative thoughts
V1 MADRS	plc	1.000000	0.488648	0.533878	0.444783	0.248330	0.772216	0.674943	0.583366	0.693911
V1 DASS	plc	0.488648	1.000000	0.800666	0.845093	0.835563	0.304479	0.325774	0.327545	0.381852
V1 D-DASS	plc	0.533878	0.800666	1.000000	0.538677	0.455306	0.384502	0.387079	0.246976	0.459478
V1 A-DASS	plc	0.444783	0.845093	0.538677	1.000000	0.586659	0.271823	0.238180	0.441911	0.235511
V1 S-DASS	plc	0.248330	0.835563	0.455306	0.586659	1.000000	0.111226	0.187176	0.144879	0.251860
V1 MADRS sadn	plc	0.772216	0.304479	0.384502	0.271823	0.111226	1.000000	0.359433	0.346940	0.363335
V1 MADRS neurc	plc	0.674943	0.325774	0.387079	0.238180	0.187176	0.359433	1.000000	-0.001203	0.496760
V1 MADRS detac	plc	0.583366	0.327545	0.246976	0.441911	0.144879	0.346940	-0.001203	1.000000	0.163394
V1 MADRS negat	plc	0.693911	0.381852	0.459478	0.235511	0.251860	0.363335	0.496760	0.163394	1.000000
V1 MADRS	pro	1.000000	0.603920	0.579986	0.536190	0.305137	0.656814	0.647747	0.683968	0.797827
V1 DASS	pro	0.603920	1.000000	0.793308	0.837565	0.754331	0.362633	0.415715	0.588406	0.447192
V1 D-DASS	pro	0.579986	0.793308	1.000000	0.499319	0.326634	0.483022	0.242507	0.556579	0.493837
V1 A-DASS	pro	0.536190	0.837565	0.499319	1.000000	0.524588	0.309046	0.476481	0.398990	0.432959
V1 S-DASS	pro	0.305137	0.754331	0.326634	0.524588	1.000000	0.036592	0.286837	0.432656	0.113191
V1 MADRS sadn	pro	0.656814	0.362633	0.483022	0.309046	0.036592	1.000000	0.218261	0.288966	0.591568
V1 MADRS neurc	pro	0.647747	0.415715	0.242507	0.476481	0.286837	0.218261	1.000000	0.237074	0.388558
V1 MADRS detac	pro	0.683968	0.588406	0.556579	0.398990	0.432656	0.288966	0.237074	1.000000	0.356643
V1 MADRS negat	pro	0.797827	0.447192	0.493837	0.432959	0.113191	0.591568	0.388558	0.356643	1.000000
